# Measurement of delayed bathing and early initiation of breastfeeding: a cross-sectional survey exploring experiences of data collectors in Ethiopia

**DOI:** 10.1186/s12887-015-0350-7

**Published:** 2015-04-08

**Authors:** Mihretab Melesse Salasibew, Girmaye Dinsa, Della Berhanu, Suzanne Filteau, Tanya Marchant

**Affiliations:** London School of Hygiene and Tropical Medicine, Keppel Street, London, WC1E 7HT UK

**Keywords:** Newborn care, Newborn behaviours, Newborn practices, Essential interventions, Delayed bathing, Breastfeeding initiation, Household survey, Data collectors, Interviewers, Probes

## Abstract

**Background:**

Delayed bathing and early initiation of breastfeeding are among the essential interventions recommended to save newborn lives. Although survey coverage reports are key to monitoring these interventions, few studies investigated whether such reports accurately reflect the proportion of mothers and children who received these interventions. In order to gather accurate data, guidance on how to interview and probe mothers is provided. In this study, we investigated experiences of data collectors when asking mothers survey questions that assessed delayed bathing and early initiation of breastfeeding.

**Methods:**

In November 2013, using a self-administered semi-structured questionnaire, we interviewed data collectors who had taken part in a population-based newborn health household survey in Ethiopia during October-November 2013. A total of 130 out of 160 invited data collectors completed and returned the self-administered questionnaire. Descriptive statistics were used to analyse quantitative data using SPSS software version 19. Qualitative data showing the variety of probes used by data collectors was analysed by listing, screening to identify common themes, and grouping by category.

**Results:**

Most data collectors reported that, in their opinion, mothers were able to understand the meaning of the question about newborn bathing (n = 102, 79%) and breastfeeding initiation (n = 106, 82%) without the need for probes. However, fewer mothers were able to recall the event for either newborn behaviours and describe it in minutes, hours or days without the need for probes. Overall, only 26% (n = 34) and 34 % (n = 44) of all data collectors reported that they did not need any probing for the questions related to newborn bathing and breastfeeding initiation questions, respectively. We identified a variety of probes used by data collectors and present examples.

**Conclusion:**

Considerable probing was necessary to facilitate maternal recall of the events and approximate their responses of time regardless of mothers’ age, level of education and parity. This could potentially lead to inaccurate coverage reports due to subjective and inconsistent interpretation of the indicators. Therefore, we recommend inclusion of standard probes or follow-on questions to the existing survey tools assessing the two indicators. Data collectors also require further guidance in using appropriate probes to gather accurate maternal responses.

**Electronic supplementary material:**

The online version of this article (doi:10.1186/s12887-015-0350-7) contains supplementary material, which is available to authorized users.

## Background

Essential interventions immediately after birth such as thermal care (immediate drying and delayed bathing), early initiation of breastfeeding (within one hour) as well as hygienic cord and skin care are recommended to save newborn lives [1-3] and there is global consensus in reaching out to more newborn babies [4]. It has been estimated that if coverage was universal, exclusive breastfeeding, thermal care and cord care could save up to 13%, 2% and 4% of all under-five deaths respectively [5]. Furthermore, up to 16% of all neonatal deaths could be prevented if all infants were breastfed from day one and 22% if breastfed within the first hour after birth [6].

Accurate measurement of essential interventions, including delayed bathing and early initiation, could inform policy makers in tracking progress made in saving newborn lives and permit comparison of coverage figures within and between countries. In many high mortality settings, tracking the extent to which every newborn benefits from these care components is limited by the lack of well-established routine health information systems. Population-based household surveys such as the Demographic and Health Survey (DHS) and the Multiple Indicator Cluster Survey (MICS) remain the main source of coverage reports based on which national and regional newborn health policies and strategic decisions are made. However, few studies have evaluated validity and reliability of such household surveys and it is unclear whether survey reports accurately reflect the proportion of mothers and children who accessed and received life-saving interventions [7].

A recent review [8] identified some challenges related to measurement of immediate care behaviours and practices for newborns, including: 1) lack of routine collection of data about immediate newborn behaviours (other than breastfeeding) during national household surveys, 2) variations in the questions asked, and 3) maternal recall and timing of specific newborn care interventions. In response to these challenges, the authors of the review recommended standardised indicators for thermal care (drying, skin-to-skin care, and delayed bathing) and hygienic skin and cord care (clean cord cutting and dry cord care; hand washing prior to delivery) be included in national household surveys, suggested that questions about immediate newborn behaviours and practices be asked of all mothers irrespective of place of birth, and also simplification of questions about timing of events.

We propose another challenge related to measurement of immediate newborn care behaviours and practices, which is the experience of the data collectors themselves. In order to gather accurate data, guidance on how to interview and probe mothers is provided to enumerators prior to data collection [9,10]. In this study, we investigated experiences of data collectors in how they asked and coded the questions assessing delayed bathing and early initiation of breastfeeding - both of which require maternal responses of time. Specifically, we report on 1) whether data collectors needed to use probes to facilitate mothers’ understanding of the questions and 2) examples of the probes used by data collectors to assist mothers’ understanding of the wording of the question, and recall of newborn events (bathing, breastfeeding initiation), including timing.

## Methods

Data were collected by inviting a large team of household survey interviewers, who were still in the field, to complete a self-administered semi-structured questionnaire and provide answers based on the most recent interview they had conducted with a mother of a newborn child.

### Setting

A population-based newborn health survey was conducted between October-November 2013 in four major regions of Ethiopia: Amhara, Oromiya, Tigray and Southern Nations, Nationalities and Peoples’ region (SNNP). The household survey, implemented as part of a measurement, learning and evaluation grant to the IDEAS project (Informed Decisions for Actions in maternal and newborn health), at London School of Hygiene and Tropical Medicine, was designed to measure population level coverage of newborn behaviours and practices, including immediate breastfeeding and delayed bathing. The survey tools were modular, consistent with the DHS structure, and included a module for women aged 13–49 years who had a recent live birth. DHS tools include a coded response ‘immediately’ but this response was not included in this survey and the wording of the question assessing early initiation, shown in Table 1, was translated to local languages. As part of the survey, eligible mothers were specifically asked about timing of breastfeeding initiation and bathing their newborn babies.

**Table 1:** **Questions assessing delayed bathing and early initiation of breastfeeding in the newborn health household survey in Ethiopia**

**Question**	**Coding**
**Delayed bathing:**	□ Within an hour, write minutes ____
“How long after birth did you first bath [name]?”	□ After an hour, write hours _______
	□ After the first day, write days ____
**Early initiation:**	□ Within an hour, write minutes ____
“How long after birth did you start breastfeeding [name]?”	□ After an hour, write hours _______
	□ After the first day, write days ____

In order to minimize language and cultural barriers, data collectors were recruited from the same region where the interviews took place and all of them had college-level or above education in health and social sciences. In addition to participating an intensive training on the data collection tool and field guide, data collectors also participated in one-day pilot testing of the tool before they started actual interviews. A total of 160 data collectors were employed for the survey and all were invited to participate in this study sharing their experiences of interviewing eligible mothers.

### Data collection

We developed a self-administered, semi-structured, paper-based questionnaire. An additional pdf file shows this in more detail [see Additional file 1: Self-administered questionnaire]. We specifically explored data collectors’ views of whether mothers understood the questions upon hearing them for the first time, and were able to recall the event and describe the timing of bathing and breastfeeding initiation in minutes, hours or days without probing or further explanation. To minimise recall bias, we asked data collectors to complete the questionnaire during the field work period and to base their responses on their experience of interviewing the most recent eligible mother. The questionnaire was translated to local languages depending on the region each data collector worked in: Amharic (Amhara and SNNP region), Tigrigna (Tigray region) and oromifa (Oromiya region).

### Data management and analysis

Completed questionnaires were returned to the central office in Addis Ababa, Ethiopia where they were double data entered using SPSS software version 19 and differences reconciled. Quantitative data was analysed using this software where mean values were calculated for continuous variables and percentages were described for categorical variables. Evidence of statistical association between maternal characteristics (age, education level, place of last birth, and parity) and reported frequency of probing by data collectors was sought using the chi square test.

Qualitative data showing examples of probes reported to have been used by data collectors were analysed by listing all reported probes in a table, screening them to identify common themes and finally grouping each probe under a specific category.

### Ethics

The study received ethical approval from the research ethics committee at the London School of Hygiene and Tropical Medicine in October 2012 and in Ethiopia from the National Research Ethics Committee under the Ministry of Science and Technology in April 2013. Participants of the study received an information sheet and a written consent form together with the questionnaire. They were asked to read these and, if they agreed, sign the consent form before completing the questionnaire. Each questionnaire was pre-assigned a number and data were analysed anonymously.

## Results

A total of 130 out of 160 invited data collectors completed and returned the questionnaire: 53 out of 72 in Oromiya, 15 out of 16 in Tigray, 26 out of 36 in SNNP and all 36 in Amhara region. The average age of the last mother the data collectors interviewed was 29 years (SD = ± 7) and most were reported to have been multiparous (n = 103, 79%), had no education (n = 89, 69%) and gave birth at home (n = 96, 74%).

### Delayed bathing

Question: How long after birth did you bath [name]?”

Most data collectors (n = 102, 79%) reported that, in their opinion, mothers were able to understand the meaning of the question upon hearing it for the first time without the need for probes. However with regards to responses about timing of when the newborn was bathed, only 39% (n = 51) and 46% (n = 60) of data collectors reported that mothers were able to recall the event and describe it in minutes, hours or days without probing, respectively. There was evidence that a higher percentage of mothers who gave birth at home were able to give responses without requiring any probing compared to mothers with a health facility delivery (31% compared to 12%, p 0.03). Overall, just over a quarter of all data collectors 26% (n = 34) reported that they did not need any probing to help mothers understand the meaning of the question, recall the event of newborn bathing, or describe it in minutes, hours or days (Table 2).

**Table 2:** **Data collector reports of mothers who understood the question, recalled and described timing for bathing babies with no probing at all**

**Maternal characteristics**	**N**	**Understood question without probes**	**Recalled timing without probes**	**Described timing without probes**	**No probing at all**	
		**n (%)**	**n (%)**	**n (%)**	**n (%)**	P-value*
**Total**	**130**	**102 (79)**	**51 (39)**	**60 (46)**	**34 (26)**	
***Age***						
15-24	36	29 (80)	18 (50)	18 (50)	12 (33)	0.29
25-34	59	47 (80)	23 (39)	28 (48)	16 (27)	
35^+^	35	26 (74)	10 (29)	14 (40)	6 (17)	
***Education***						
No education	89	66 (74)	31 (35)	37 (42)	19 (21)	0.07
Primary level or above	41	36 (88)	20 (49)	23 (56)	15 (37)	
***Place of birth***						
Home	96	76 (79)	42 (44)	45 (47)	30 (31)	0.03
Health institution	34	26 (77)	9 (27)	15 (44)	4 (12)	
***Parity***						
Primiparous	27	22 (82)	14 (52)	16 (59)	10 (37)	0.15
Multiparous	103	80 (78)	37 (36)	44 (43)	24 (23)	

*P values were obtained from chi-square tests.

### Early initiation of breastfeeding

Question: How long after birth did [name] start breastfeeding?”

Most data collectors (n = 106, 82%) reported that, in their opinion, mothers understood the question about breastfeeding initiation upon hearing it for the first time without the need for probes. However, only 48% (n = 62) reported that they didn’t need to probe mothers both to help them recall the event of breastfeeding initiation or describe the time in minutes, hours or days. Overall, only a third of all data collectors 34% (n = 44) reported that they did not need any probing to help mothers understand the meaning of the question, recall the event of breastfeeding initiation or describe it in minutes, hours or days (Table 3).

**Table 3:** **Data collector reports of mothers who understood the question, recalled and described timing for breastfeeding initiation with no probing at all**

**Maternal characteristics**	**N**	**Understood question without probes**	**Recalled timing without probes**	**Described timing without probes**	**No probing at all**	
		**n (%)**	**n (%)**	**n (%)**	**n (%)**	P-value*
**Total**	**130**	**106 (82)**	**62 (48)**	**62 (48)**	**44 (34)**	
***Age***						
15-24	36	31 (86)	22 (61)	20 (56)	16 (44)	
25-34	59	49 (83)	27 (46)	27 (46)	19 (32)	0.23
35^+^	35	26 (74)	13 (37)	15 (43)	9 (26)	
***Education***						
No education	89	69 (78)	37 (42)	35 (39)	26 (29)	0.10
Primary level or above	41	37 (90)	25 (61)	27 (66)	18 (44)	
***Place of birth***						
Home	96	78 (81)	50 (52)	48 (50)	36 (38)	0.14
Health institution	34	28 (82)	12 (35)	14 (41)	8 (24)	
***Parity***						
Primiparous	27	23 (85)	17 (63)	14 (52)	12 (44)	0.19
Multiparous	103	83 (81)	45 (44)	48 (47)	32 (31)	

*P values were obtained from chi-square tests.

### Probes

Data collectors reported using a variety of probes to help mothers understand the questions about newborn bathing and breastfeeding initiation, recall the event, and describe timing in minutes, hours or days. We identified three groups of probes used by data collectors: 1) probes which simply re-phrased the wording of the question, 2) probes that used some reference points during birth such as delivery of placenta, and 3) probes that sequentially listed all events surrounding birth in order to facilitate maternal recall.

Examples of probes which simply re-phrased the wording or text description of the questions include the following.

*“From the time you gave birth, when was the first time the baby come in contact with water?” (Male data collector, degree level qualified, Amhara region)*

*“As per the advice from health extension workers, did you delay bathing your baby to prevent baby getting cold and by how long?” (Male data collector, degree level qualified, Oromiya region)*

*“How many minutes or hours after (name) was born did you get her to breastfeed?” (Male data collector, diploma level qualified, SNNP region)*

*“Did he breastfeed as soon as you gave birth? Or did the baby breastfeed on the same day of his birth, or the second day, or the following consecutive days?” (Female data collector, degree level qualified, SNNP region)*

Probes which used reference points of an event around birth in order to help mothers recall the event and describe the time include the following.

*“How long after the placenta was delivered did you bath your baby?” (Female data collector, Nurse, SNNP region)*

*“When you bath your baby for the first time after birth, was it breakfast time, lunch, dinner or time when the cattle came back home?” (Female data collector, diploma level qualified, SNNP region)*

*“When did you start breastfeeding your baby? Was it before the placenta came out or after? If it is, how long after delivery of the placenta did you start breastfeeding?” (Female data collector, diploma level qualified, SNNP region)*

*“Did you start breastfeeding after the placenta came out and you were given kacha (traditional meal given to mothers soon after birth)? How long after birth was that?” (Female data collector, degree level qualified, SNNP region)*

Finally, probes which were used to ask mothers sequentially going through all events following birth in order to estimate approximate time for bathing the baby include the following.

*“What happened after the baby was born and placenta delivered? Did they dry the baby and wrap him in a cloth? What time did they bath him?” (Male data collector, degree level qualified, Amhara region)*

*“Who attended the birth? Did they bath your baby? Where was the baby kept after that and when did they give you your baby to start breastfeeding?” (Male data collector, degree level qualified, Amhara region)*

*“Did they bath your baby at the health center or was it after you went back home? How long did you stay in the health center after giving birth? Did you start breastfeeding in the health center or once you got back home? How long after birth could this be?” (Male data collector, degree level qualified, Tigray region)*

## Discussion

Although most data collectors reported that the majority of mothers they interviewed understood the meaning of the essential newborn care questions, considerable probing was still needed to facilitate recall and approximate responses of time regardless of maternal age, educational level, parity and place of delivery. We also identified variation in the types of probes that the data collectors used.

A limitation of this study was whether data collectors accurately recalled and reported their experience of interviewing mothers using the self-administered questionnaires provided. Attempts were made to minimize recall bias, for example by asking them to base their answers on the most recent interviews, and by providing them information sheet and orientation in order to help them accurately complete the questionnaire. Nonetheless, data collectors might have chosen to share their experiences based on the “best or worst” interviewee they had and therefore, their perception of how mothers understood the questions could still be inaccurate.

Despite this limitation, some consistent results emerged. According to data collector reports, although most mothers were clear about the wording or text descriptions of both questions, considerable probing was necessary to facilitate maternal recall of the event and approximate their responses of time as per the recommended coding format. Consistent with a study in South Africa [11], we found no statistical evidence of association between mothers’ characteristics (age, education or parity) and their ability to understand the questions, recall the event and describe timing without any probing.

However, we did observe that mothers who gave birth at home could answer questions without probing more frequently than mothers who gave birth in a health facility. This could partly be explained by limited involvement of mothers with immediate newborn care practices due to hospital protocols even when they had non-emergency deliveries, and therefore mothers may need more prompting to remember the sequence of events. We also identified different examples of probes used as follow-on questions for mothers who needed clarification about the questions. Currently, there are no standard probes or follow-on questions recommended in household surveys such as DHS [9,12] and MICS [10,13] to avoid leading questions or subjective and possibly inaccurate interpretations of questions assessing the two time-dependent newborn indicators i.e. delayed bathing and early initiation of breastfeeding.

Accuracy in self-reports of mothers who participated in population-based household surveys have been assessed [14,15] and recommendations made to improve measurement and reporting of coverage estimates [8]. Accuracy of survey data can depend on a number of factors including the length of recall period [16-18], demographic characteristics of respondents [11] inter- or intra-observer variations [19,20], social desirability i.e. reporting about own practice based on socially acceptable norms [21-23] and approaches to data collection [21-24] In this study, we have revealed variations in how data collectors interviewed and probed mothers to explain time-dependent questions about newborn bathing and breastfeeding initiation.

## Conclusion

Data collectors reported that considerable probing was necessary to facilitate maternal recall of the event and approximate their responses of time regardless of mothers’ age, level of education and parity. This could potentially lead to inaccurate coverage reports due to subjective and inconsistent interpretation of the indicators, which makes it difficult to track progress in the implementation of newborn life-saving interventions. Therefore, we recommend inclusion of standard probes or follow-on questions to the existing survey tools assessing the two newborn indicators. Data collectors also require further guidance about the purpose and interpretation of the indicators as well using appropriate probes to gather accurate maternal responses.

### Availability of supporting data

The data set is published on LabArchives with DOI: 10.6070/H4SQ8XDD.

## Additional file

Additional file 1:
**Self-administered questionnaire.** This file shows the 13 questions we asked data collectors in order to explore their experiences of asking mothers survey questions that assessed delayed bathing and early initiation of breastfeeding.

## Abbreviations

DHS: Demographic and Health Survey
MICS: Multiple Indicator Cluster Survey
SNNP: Southern Nations, Nationalities and Peoples’ region
IDEAS: Informed Decisions for Actions in maternal and newborn health
