# Supplementary material for: Measurement of delayed bathing and early initiation of breastfeeding: a cross-sectional survey exploring experiences of data collectors in Ethiopia
Source: BMC Pediatr. 2015 Apr 8;15:35. doi: 10.1186/s12887-015-0350-7 (PMC4391328; doi:10.1186/s12887-015-0350-7)
Supplement: Additional file 1: — Self-administered questionnaire. This file shows the 13 questions we asked data collectors in order to explore their experiences of asking mothers survey questions that assessed delayed bathing and early initiation of breastfeeding. [file 12887_2015_350_MOESM1_ESM.pdf]

## Self-administered questionnaire

**Informed consent**

This survey, led by **Mihretab M Salasibew**, is being conducted as part of a PhD research on Measurement of new born behaviours and practices in Ethiopia. **Della Berhanu** and **Girmaye Dinsa** from London School of Hygiene and Tropical Medicine in the UK will lead data collection process.

The survey aims at assessing your experiences in asking questions on **Module 3, section 8 of the IDEAS newborn health household survey**. The questionnaire is self-administered and is expected to take not more than 10 minutes of your time. The survey is being conducted anonymously and data will be analysed without reference to your name or the name of the organization you represent or worked for. Data will be accessed only by the primary investigator and all findings from this survey will solely be used for the purpose of this PhD research. There are no known risks to you by participating in this survey and there are no direct benefits to you. However, outcomes of this survey will help in improving measurement of new born behaviours and practices in Ethiopia. Taking part in this survey is your choice. If you feel uncomfortable about any of the questions, you can refuse to answer. If you decide not to participate in this survey, you may not lose any services that you are otherwise receiving. Any questions about this survey, you may call **Della Berhanu** or **Girmaye Dinsa** on ____________________________.

If you are happy to participate in this survey, you are kindly asked to give your consent by signing below and complete the following questions.

Signature: ____________ Date: Day ________, Month _______, Year 2006 E.C

| S/N | Question | Response |
|---|---|---|
| | **ABOUT YOU** | |
| 1. | Gender __________ **(Please circle your answer)** | ☐ Male ☐ Female |
| 2. | Main occupation ________ **(Please write your answer)** | My main occupation or job is _____________ |
| 3. | Education ____ _______ **(please write your answer)** | The highest level of education I achieved is ____________________ |
| 4. | Where do you normally live?<br>**(Please write the woreda or city, and Region you live )** | I live in ___________________ |
| 5. | Which language (s) can you speak?<br>**(Please circle all that apply)** | ☐Amharic ☐ Oromigna<br>☐ Guragigna ☐ Woliyitigna<br>☐Tigrigna ☐ Sidamigna<br>☐ Other (please specify)<br>_______________ |

| Please think about the last mother you interviewed on Module 3, section 8 of the IDEAS newborn health household questionnaire and answer the following | | |
|---|---|---|
| | | |
| 6. | In which region of Ethiopia was that interview carried out? **(Please circle one answer)** | ☐ Amhara ☐ Tigray<br>☐ Oromiya ☐ SNNP<br>☐ Other (Please specify) __________ |
| 7. | In which month and year was that interview carried out? For example, if it was in February 2004 E.C, enter 06/2004. | ☐ Month _______<br>☐ Year __________E.C |
| 8. | How old is the mother? **(Please write your answer)** | The mother is _______years old |
| 9. | What is her level of education? **(Please circle one answer)** | a) Illiterate<br>b) Elementary school or higher |
| 10 | Where did the mother give her last birth? **(Please circle one answer)** | a) Home<br>b) Health institution |
| 11 | How many live births has the mother had including the last birth? **(Please circle one answer)** | a) This is her first child<br>b) More than one child |
| 12 | Questions 825-- 828 asked how long after birth did the mother bath her baby. When you first asked this mother this question, | |
| | a) Would you say the mother understood the question **on first attempt**? | ☐ YES ☐ NO |
| | b) Did the mother need more explanation in order to help her **recall**? | ☐ YES ☐ NO |
| | c) Did the mother need more explanation about how to **express time** in minutes, hours or days? | ☐ YES ☐ NO |
| | d) If the mother needed more explanation about bathing recall or to express time, what | |

| | | |
|---|---|---|
| | explanations did you provide? (**Please write you explanation)** | |
| 13 | Question 837 asked how long after birth did the mother start breastfeeding her baby. When you first asked this question, | |
| | a) Would you say the mother understood the question **on first attempt**? **(please circle one answer)** | ☐ YES ☐ NO |
| | b) Did the mother need more explanation in order to help her **recall**? **(please circle one answer)** | ☐ YES ☐ NO |
| | c) Did the mother need more explanation about how to **express time** in minutes, hours or days? **(please circle one answer)** | ☐ YES ☐ NO |
| | d) If the mother needed more explanation about breastfeeding recall or to express time, what explanations did you provide? (**Please write you explanation)** | |

Thank you for your time in completing the questionnaire

Please **RETURN** the completed questionnaire to either Della Berhanu or Girmaye Dinsa.
